# Low-Complexity Alternatives to the Optimal Linear Coding Scheme for Transmitting ARMA Sources

**DOI:** 10.3390/e24050669

**Published:** 2022-05-10

**Authors:** Jesús Gutiérrez-Gutiérrez, Fernando M. Villar-Rosety, Xabier Insausti, Marta Zárraga-Rodríguez

**Affiliations:** Department of Biomedical Engineering and Sciences, Tecnun, University of Navarra, Manuel Lardizábal 13, 20018 Donostia-San Sebastián, Spain; jgutierrez@tecnun.es (J.G.-G.); xinsausti@tecnun.es (X.I.); mzarraga@tecnun.es (M.Z.-R.)

**Keywords:** low-complexity, analog linear coding, short packet communication, finite-length data blocks, AR sources, MA sources, ARMA sources, WSS sources, DFT

## Abstract

In the era of the Internet of Things, there are many applications where numerous devices are deployed to acquire information and send it to analyse the data and make informed decisions. In these applications, the power consumption and price of the devices are often an issue. In this work, analog coding schemes are considered, so that an ADC is not needed, allowing the size and power consumption of the devices to be reduced. In addition, linear and DFT-based transmission schemes are proposed, so that the complexity of the operations involved is lowered, thus reducing the requirements in terms of processing capacity and the price of the hardware. The proposed schemes are proved to be asymptotically optimal among the linear ones for WSS, MA, AR and ARMA sources.

## 1. Introduction

In the past few years, and specially in the context of the Internet of Things (IoT), numerous applications in which devices with constrained power and processing capacity acquire and transmit data have emerged. Therefore, it is crucial to find coding strategies for transmitting information with an acceptable distortion that are undemanding in terms of processing while minimizing power consumption. A well-known low-power and low-complexity coding strategy is the so called analog joint source-channel coding (see, e.g., [1]). In this context, some authors have lately proposed the use of low-power/low-cost all-analog sensors [2,3], avoiding the high power demanding analog-to-digital converters (ADC).

Here, we are focused on analog joint source-channel linear coding. Specifically, we study here the transmission of realizations of an *n*-dimensional continuous random vector over an additive white Gaussian noise (AWGN) channel employing a linear encoder and decoder. For this scenario, the minimum average transmission power under a fixed average distortion constraint is achieved whenever it is applied the coding strategy given by Lee and Petersen in [4]. Nevertheless, in [5,6] it was presented a linear coding scheme based on the discrete Fourier transform (DFT) that, with a lower complexity, is asymptotically optimal among the linear schemes in terms of transmission power for certain sources, namely wide-sense stationary (WSS) and asymptotically WSS (AWSS) autoregressive (AR) sources.

In this paper, we present two new DFT-based alternatives to the optimal linear coding scheme given in [4]. We prove that the average transmission power required for one of them is lower than for the approach in [5,6]. The average transmission power required for the other new alternative is shown to be higher, although it is conceptually simpler, and so is its implementation. We prove that the two new schemes along with the strategy in [5,6] are asymptotically optimal for any AWSS source. Additionally, we show that the convergence speed of the average transmission power of the alternative schemes is $O(1/\sqrt{n})$ for the case of WSS, moving average (MA), AWSS AR and AWSS autoregressive moving average (ARMA) sources. Therefore, we conclude that it is possible to achieve a good performance with small size data blocks, allowing this schemes to be used in applications that require low latency.

This paper is organized as follows. In Section 2, we mathematically describe the communications system and review the transmission strategy presented in [4], and the alternative given in [5,6]. In Section 3, the two new coding schemes are presented. In Section 4, we compare the performance of each of the four coding strategies studied in terms of the required transmission power, and we analyze their asymptotic behavior for several types of sources. In Section 5 and Section 6 a numerical example and some conclusions are presented respectively.

## 2. Preliminaries

We begin this section by introducing some notation. We will denote by R, C, Z and N the sets of real numbers, complex numbers, integers and positive integers respectively. ⌈a⌉ represents the smallest integer higher than or equal to a∈R. $R^{m\times n}$ is the set of the m×n real matrices. The symbols ⊤ and ∗ denote transpose and conjugate transpose respectively, the imaginary unit is represented by i, E stands for expectation and δ is the Kronecker delta (i.e., $\delta_{j,k}=1$ if j=k, and it is zero otherwise). If z∈C, Re(z) and Im(z) will designate the real part and the imaginary part of *z* respectively, $\bar{z}$ is the conjugate of *z* and $\hat{z}$ is 2-dimensional the real column vector Re(z),Im(z)⊤. $I_{n}$ denotes the n×n identity matrix and $V_{n}$ is the n×n Fourier unitary matrix, i.e.,

$${\left[V_{n}\right]}_{j,k}=\frac{1}{\sqrt{n}}e^{-\frac{2\pi (j-1)(k-1)}{n}i}\;j,k\in \left\{1,\ldots ,n\right\}.$$

$\lambda_{1}\left(A\right),\ldots ,\lambda_{n}\left(A\right)$ and $\sigma_{1}\left(B\right),\ldots ,\sigma_{n}\left(B\right)$ are the eigenvalues of an n×n real symmetric matrix *A* and the singular values of an n×n matrix *B* sorted in decreasing order, respectively. If ${\left\{x_{n}\right\}}_{n\in N}$ is a random process, we denote by $x_{n:1}$ the random *n*-dimensional column vector ${\left(x_{n},x_{n-1},\ldots ,x_{1}\right)}^{⊤}$. The Frobenius norm and the spectral norm are represented by ${∥\cdot ∥}_{F}$ and ${∥\cdot ∥}_{2}$, respectively.

If f:R→C is a continuous and 2π-periodic function, we denote by $T_{n}\left(f\right)$ the n×n Toeplitz matrix given by

$${\left[T_{n}\left(f\right)\right]}_{j,k}=f_{j-k},\;j,k\in \left\{1,\ldots ,n\right\},$$

where ${\left\{f_{k}\right\}}_{k\in Z}$ is the set of Fourier coefficients of *f*:

$$f_{k}=\frac{1}{2\pi }\int_{0}^{2\pi }f\left(\omega \right)e^{-k\omega i}d\omega \;\forall k\in Z.$$

### 2.1. Problem Statement

We consider a discrete-time analog *n*-dimensional real vector source $x_{n:1}$ with invertible correlation matrix. We want to transmit realizations of this vector by using *n* times a real AWGN channel with noise variance $\sigma^{2}>0$. The *n* noise iid random variables can then be represented by the Gaussian *n*-dimensional vector $\nu_{n:1}$, with correlation matrix $E\left(\nu_{n:1}\nu_{n:1}^{⊤}\right)=\sigma^{2}I_{n}$. We further assume that the input $x_{n:1}$ and the channel noise $\nu_{n:1}$ are both zero-mean and that they are uncorrelated, i.e., $E(x_{j}\nu_{k})=0$ for all j,k∈{1,…,n}.

**Figure 1:**
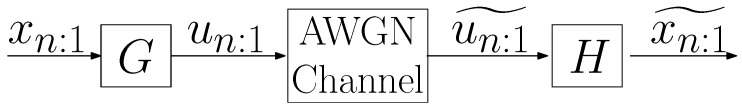
Linear coding scheme.

The communications system is depicted in Figure 1, where *G* and *H* are n×n real matrices representing the linear encoder and decoder, respectively. Specifically, a source vector symbol is encoded using a linear transformation, $u_{n:1}=Gx_{n:1}$, and then transmitted through the AWGN channel. An estimation of the source vector symbol is then obtained from the perturbed vector, $\tilde{u_{n:1}}=u_{n:1}+\nu_{n:1}$, using another linear transformation, $\tilde{x_{n:1}}=H\tilde{u_{n:1}}$.

In [4], Lee and Petersen found matrices *G* and *H* that minimize the average transmission power, $\frac{1}{n}\sum_{j=1}^{n}E\left(u_{j}^{2}\right)$, under a given average distortion constraint *D*, that is,

$$\frac{1}{n}E\left({∥x_{n:1}-\tilde{x_{n:1}}∥}_{F}^{2}\right)\leq D.$$

### 2.2. Known Linear Coding Schemes

#### 2.2.1. Optimal Linear Coding Scheme

**Figure 2:**
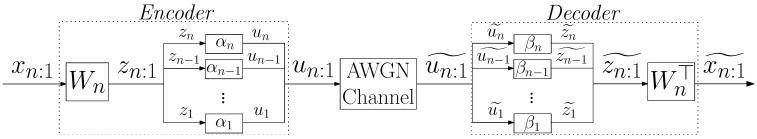
Linear coding scheme based on an n×n real orthogonal matrix $W_{n}$.

As it has been aforementioned, Lee and Petersen presented the optimal linear coding scheme in [4]. The encoder of the optimal linear coding scheme converts the *n* correlated variables of the source vector into *n* uncorrelated variables, and assigns a weight to each of the *n* uncorrelated variables. If $E\left(x_{n:1}x_{n:1}^{⊤}\right)=U_{n}\mathrm{diag}(\lambda_{1}\left(E\left(x_{n:1}x_{n:1}^{⊤}\right)\right),\ldots ,$ $\lambda_{n}\left(E\left(x_{n:1}x_{n:1}^{⊤}\right)\right))U_{n}^{-1}$ is an eigenvalue decomposition of the correlation matrix of the source, where the eigenvector matrix $U_{n}$ is real and orthogonal, the optimal linear coding scheme is of the type shown in Figure 2, with $W_{n}=U_{n}^{⊤}$. Its average transmission power is

(1) $$P_{n}\left(D\right)=\frac{\sigma^{2}}{D}\left({\left(\frac{1}{n}\sum_{j=1}^{n}\sqrt{\lambda_{j}\left(E\left(x_{n:1}x_{n:1}^{⊤}\right)\right)}\right)}^{2}-D\right)$$

under a given average distortion constraint $D\in \left(0,\lambda_{n}\left(E\left(x_{n:1}x_{n:1}^{⊤}\right)\right)\right]$.

Since this coding scheme implies a multiplication of an n×n matrix by an n×1 column vector, its computational complexity is $O(n^{2})$.

#### 2.2.2. DFT-Based Alternative

In [5,6], an alternative to the optimal coding scheme was presented. The encoder of that alternative scheme assigns weights to the real and imaginary parts of the entries of the DFT of the source vector. This alternative coding scheme is of the type shown in Figure 2, with $W_{n}=M_{n}V_{n}^{*}$, where $M_{n}$ is the n×n sparse matrix defined in ([5] Equation (3)). Its average transmission power is

(2) $${\hat{P}}_{n}\left(D\right)=\frac{\sigma^{2}}{D}\left({\left(\frac{1}{n}\sum_{j=1}^{n}\sqrt{E\left(z_{j}^{2}\right)}\right)}^{2}-D\right)$$

under a given average distortion constraint $D\in \left(0,\lambda_{n}\left(E(x_{n:1}x_{n:1}^{⊤})\right)\right]$, where

Ezn−j+12=Eyn−j+12ifj∈1,n2+1∩N,2EReyn−j+12if2≤j≤⌈n2⌉,2EImyn−j+12ifn−⌈n2⌉+2≤j≤n,

with $y_{n:1}=V_{n}^{*}x_{n:1}$.

The computational complexity of this coding scheme is O(nlog(n)) whenever the fast Fourier transform (FFT) algorithm is used.

## 3. New Coding Schemes

In this section, we propose two new transmission schemes. Similarly to the scheme reviewed in Section 2.2.2, our two new schemes make use of the DFT, and their computational complexity is also O(nlog(n)) if the FFT algorithm is applied.

### 3.1. Low-Power Alternative

**Figure 3:**
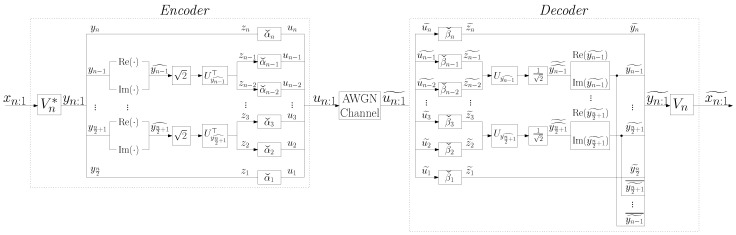
Low-power scheme.

The encoder of this scheme first computes the DFT of the source vector, $y_{n:1}=V_{n}^{*}x_{n:1}$. Afterwards, each 2-dimensional vector $\hat{y_{j}}$ is encoded using a 2×2 real orthogonal eigenvector matrix of the correlation matrix $E\left(\hat{y_{j}}{\hat{y_{j}}}^{⊤}\right)$. Therefore, the real part and the imaginary part of each $y_{j}$ are here jointly encoded, unlike in Section 2.2.2, where they where separately encoded. This coding scheme is shown in Figure 3 for *n* even (the scheme for *n* odd is similar), with

$$E\left(\hat{y_{j}}{\hat{y_{j}}}^{⊤}\right)\;=\;U_{\hat{y_{j}}}\mathrm{diag}\;\left(\lambda_{1}\;\left(E\left(\hat{y_{j}}{\hat{y_{j}}}^{⊤}\right)\right)\;,\lambda_{2}\;\left(E\left(\hat{y_{j}}{\hat{y_{j}}}^{⊤}\right)\right)\right)U_{\hat{y_{j}}}^{-1}\;\forall j\in \left\{\left\lceil \frac{n+1}{2}\right\rceil ,\ldots ,n-1\right\}$$

being an eigenvalue decomposition of $E\left(\hat{y_{j}}{\hat{y_{j}}}^{⊤}\right)$ where the eigenvector matrix $U_{\hat{y_{j}}}$ is real and orthogonal,

(3) $${\overset{ˇ}{\alpha }}_{j}=\sqrt{\frac{1}{E(z_{j}^{2})}\left(\sqrt{\frac{\sigma^{2}\left(\sigma^{2}+{\overset{ˇ}{P}}_{n}\left(D\right)\right)E\left(z_{j}^{2}\right)}{D}}-\sigma^{2}\right)}\;\forall j\in \left\{1,\ldots ,n\right\}$$

and

(4) $${\overset{ˇ}{\beta }}_{j}=\frac{{\overset{ˇ}{\alpha }}_{j}E\left(z_{j}^{2}\right)}{{\overset{ˇ}{\alpha }}_{j}^{2}E\left(z_{j}^{2}\right)+\sigma^{2}}\;\forall j\in \left\{1,\ldots ,n\right\}.$$

In Appendix A, we prove that the average transmission power of this scheme under a given average distortion constraint *D* is

(5) $${\overset{ˇ}{P}}_{n}\left(D\right)=\frac{\sigma^{2}}{D}\left({\left(\frac{1}{n}\sum_{j=1}^{n}\sqrt{E\left(z_{j}^{2}\right)}\right)}^{2}-D\right)\;\forall D\in \left(0,\lambda_{n}\left(E\left(x_{n:1}x_{n:1}^{⊤}\right)\right)\right],$$

with

$$E\left(z_{n-j+1}^{2}\right)=\left\{\begin{array}{cc} E\left(y_{n}^{2}\right) & \mathrm{if}\;j=1,\\ 2\lambda_{1}\left(E\left(\hat{y_{n-\frac{j}{2}}}{\hat{y_{n-\frac{j}{2}}}}^{⊤}\right)\right) & \mathrm{if}\;j\;\mathrm{even},\;j\neq n,\\ 2\lambda_{2}\left(E\left(\hat{y_{n-\frac{j-1}{2}}}{\hat{y_{n-\frac{j-1}{2}}}}^{⊤}\right)\right) & \mathrm{if}\;j\;\mathrm{odd},\;j\neq 1,\\ E\left(y_{\frac{n}{2}}^{2}\right) & \mathrm{if}\;j=n,\;n\;\mathrm{even}. \end{array}\right.$$

Furthermore, in Section 4, we show that ${\overset{ˇ}{P}}_{n}\left(D\right)\leq {\hat{P}}_{n}\left(D\right)$, i.e., the average transmission power for this new scheme is lower than the one required for the scheme in Section 2.2.2.

### 3.2. DFT/IDFT Alternative

**Figure 4:**
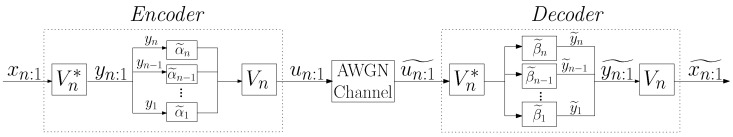
DFT/IDFT scheme.

The encoder of this scheme uses both the DFT and the inverse DFT (IDFT). The coding scheme is shown in Figure 4, where

(6) $${\tilde{\alpha }}_{j}=\sqrt{\frac{1}{E\left({\left|y_{j}\right|}^{2}\right)}\left(\sqrt{\frac{\sigma^{2}\left(\sigma^{2}+{\tilde{P}}_{n}\left(D\right)\right)E\left({\left|y_{j}\right|}^{2}\right)}{D}}-\sigma^{2}\right)}\;\forall j\in \left\{1,\ldots ,n\right\}$$

and

(7) $${\tilde{\beta }}_{j}=\frac{{\tilde{\alpha }}_{j}E\left({\left|y_{j}\right|}^{2}\right)}{{\tilde{\alpha }}_{j}^{2}E\left({\left|y_{j}\right|}^{2}\right)+\sigma^{2}}\;\forall j\in \left\{1,\ldots ,n\right\}.$$

In Appendix B, we prove that the average transmission power of this scheme under a given average distortion constraint *D* is

(8) $${\tilde{P}}_{n}\left(D\right)=\frac{\sigma^{2}}{D}\left({\left(\frac{1}{n}\sum_{j=1}^{n}\sqrt{E\left({\left|y_{j}\right|}^{2}\right)}\right)}^{2}-D\right)\;\forall D\in \left(0,\lambda_{n}\left(E\left(x_{n:1}x_{n:1}^{⊤}\right)\right)\right].$$

As it can be seen in Figure 4, this coding scheme is conceptually simpler than the ones in Section 2.2.2 and Section 3.1.

## 4. Analysis of the Transmission Power of the Coding Schemes

We begin this section by comparing the performance of each of the four considered coding strategies in terms of the required transmission power.

**Theorem** **1.** *Let $P_{n}\left(D\right)$, ${\hat{P}}_{n}\left(D\right)$, ${\overset{ˇ}{P}}_{n}\left(D\right)$ and ${\tilde{P}}_{n}\left(D\right)$ be the average transmission powers given in (1), (2), (5) and (8), respectively. Then,*

(9) $$P_{n}\left(D\right)\leq {\overset{ˇ}{P}}_{n}\left(D\right)\leq {\hat{P}}_{n}\left(D\right)\leq {\tilde{P}}_{n}\left(D\right).$$

**Proof.**  See Appendix C. □

### 4.1. Asymptotic Behavior

We now show that the three alternatives to the optimal linear coding scheme, which were presented in Section 2.2.2, Section 3.1 and Section 3.2, are asymptotically optimal whenever the source is AWSS. To that end, we first need to review three definitions. We begin with the Gray concept of asymptotically equivalent sequences of matrices given in [7].

**Definition** **1** **(Asymptotically equivalent sequences of matrices).** *Let $A_{n}$ and $B_{n}$ be n×n matrices for all n∈N. The two sequences of matrices ${\left\{A_{n}\right\}}_{n\in N}$ and ${\left\{B_{n}\right\}}_{n\in N}$ are said to be asymptotically equivalent, abbreviated $\left\{A_{n}\right\}∼\left\{B_{n}\right\}$, if there exists M≥0 such that*

$$∥A_{n}{∥}_{2},{∥B_{n}∥}_{2}\leq M\;\forall n\in N$$

*and*

$$\lim_{n\to \infty }\frac{∥A_{n}-B_{n}{∥}_{F}}{\sqrt{n}}=0.$$

Now we recall the well-known concept of WSS process.

**Definition** **2** **(WSS process).** Let f:R→R be a continuous, 2π-periodic and non-negative function. A random process ${\left\{x_{n}\right\}}_{n\in N}$ is said to be WSS, with power spectral density (PSD) f, if it has constant mean, i.e., $E\left(x_{j}\right)=E\left(x_{k}\right)\;\forall j,k\in N$, and $\left\{E\left(x_{n:1}x_{n:1}^{*}\right)\right\}=\left\{T_{n}\left(f\right)\right\}$.

We can now review the Gray concept of AWSS process given in ([8] p. 225).

**Definition** **3** **(AWSS process).** Let f:R→R be a continuous, 2π-periodic and non-negative function. A random process ${\left\{x_{n}\right\}}_{n\in N}$ is said to be AWSS, with (asymptotic) PSD f, if it has constant mean and $\left\{E\left(x_{n:1}x_{x:1}^{*}\right)\right\}∼\left\{T_{n}\left(f\right)\right\}$.

**Theorem** **2.** *Suppose that ${\left\{x_{n}\right\}}_{n\in N}$ is an AWSS process with PSD f as in Definition 3. If $\underset{n\in N}{\inf}\lambda_{n}\left(E\left(x_{n:1}x_{n:1}^{⊤}\right)\right)>0$ then*

$$\lim_{n\to \infty }P_{n}\left(D\right)\;=\;\lim_{n\to \infty }{\hat{P}}_{n}\left(D\right)\;=\;\lim_{n\to \infty }{\overset{ˇ}{P}}_{n}\left(D\right)\;=\;\lim_{n\to \infty }{\tilde{P}}_{n}\left(D\right)\;=\;\frac{\sigma^{2}}{D}\left(\;{\left(\frac{1}{2\pi }\int_{0}^{2\pi }\sqrt{f(\omega )}d\omega \right)}^{2}-D\;\right)$$

*for all $D\in \left(0,\underset{n\in N}{\inf}\lambda_{n}\left(E(x_{n:1}x_{n:1}^{⊤})\right)\right]$.*

**Proof.**  See Appendix D. □

### 4.2. Convergence Speed

Here, we prove that the convergence speed of the average transmission power of the three alternative schemes described in Section 2.2.2, Section 3.1 and Section 3.2 to the average transmission power of the optimal linear scheme is $O(1/\sqrt{n})$ for AWSS ARMA, MA, AWSS AR and WSS sources. We also recall the definitions of ARMA, MA and AR processes.

#### 4.2.1. AWSS ARMA Sources

**Definition** **4** **(ARMA process).** *A real zero-mean random process ${\left\{x_{n}\right\}}_{n\in N}$ is said to be ARMA if*

(10) $$x_{n}=w_{n}+\sum_{j=1}^{n-1}b_{-j}w_{n-j}-\sum_{j=1}^{n-1}a_{-j}x_{n-j}\;\forall n\in N,$$

*where $b_{-j},a_{-j}\in R$ for all j∈N, and ${\left\{w_{n}\right\}}_{n\in N}$ is a real zero-mean random process satisfying that $E\left(w_{j}w_{k}\right)=\delta_{j,k}\sigma_{w}^{2}$ for all j,k∈N with $\sigma_{w}^{2}>0$. If there exist p,q∈N such that $a_{-j}=0$ for all j>p and $b_{-j}=0$ for all j>q, then ${\left\{x_{n}\right\}}_{n\in N}$ is called an ARMA(p,q) process.*

**Theorem** **3.** *Suppose that ${\left\{x_{n}\right\}}_{n\in N}$ is an ARMA(p,q) process as in Definition 4. Let $a\left(\omega \right)=1+\sum_{j=1}^{p}a_{-j}e^{-j\omega i}$ and $b\left(\omega \right)=1+\sum_{j=1}^{q}b_{-j}e^{-j\omega i}$ for all ω∈R. Assume that a(ω)≠0 and b(ω)≠0 for all ω∈R, and that there exist $K_{1},K_{2}>0$ such that ${∥{\left(T_{n}\left(a\right)\right)}^{-1}∥}_{2}\leq K_{1}$ and ${∥{\left(T_{n}\left(b\right)\right)}^{-1}∥}_{2}\leq K_{2}$ for all n∈N. Then, ${\left\{x_{n}\right\}}_{n\in N}$ is AWSS with PSD $\sigma_{w}^{2}\frac{{\left|b\right|}^{2}}{{\left|a\right|}^{2}}$, and*

(11) $${\tilde{P}}_{n}\left(D\right)-P_{n}\left(D\right)=O\left(1/\sqrt{n}\right).$$

*for all $D\in \left(0,\underset{n\in N}{\inf}\lambda_{n}\left(E(x_{n:1}x_{n:1}^{⊤})\right)\right]$.*

**Proof.**  See Appendix E. □

#### 4.2.2. MA Sources

**Definition** **5** **(MA process).** *A real zero-mean random process ${\left\{x_{n}\right\}}_{n\in N}$ is said to be MA if*

$$x_{n}=w_{n}+\sum_{j=1}^{n-1}b_{-j}w_{n-j}\;\forall n\in N,$$

*where $b_{-j}\in R$ for all j∈N, and ${\left\{w_{n}\right\}}_{n\in N}$ is a real zero-mean random process satisfying that $E\left(w_{j}w_{k}\right)=\delta_{j,k}\sigma_{w}^{2}$ for all j,k∈N with $\sigma_{w}^{2}>0$. If there exists q∈N such that $b_{-j}=0$ for all j>q, then ${\left\{x_{n}\right\}}_{n\in N}$ is called an MA(q) process.*

**Theorem** **4.** *Suppose that ${\left\{x_{n}\right\}}_{n\in N}$ is an MA(q) process as in Definition 5. Let $b\left(\omega \right)=1+\sum_{j=1}^{q}b_{-j}e^{-j\omega i}$ for all ω∈R. Assume that b(ω)≠0 for all ω∈R, and that there exists K>0 such that ${∥{\left(T_{n}\left(b\right)\right)}^{-1}∥}_{2}\leq K$ for all n∈N. Then, ${\left\{x_{n}\right\}}_{n\in N}$ is AWSS with PSD $\sigma_{w}^{2}{\left|b\right|}^{2}$, and*

$${\tilde{P}}_{n}\left(D\right)-P_{n}\left(D\right)=O\left(1/\sqrt{n}\right).$$

*for all $D\in \left(0,\underset{n\in N}{\inf}\lambda_{n}\left(E(x_{n:1}x_{n:1}^{⊤})\right)\right]$.*

**Proof.**  It is a direct consequence of Theorem 3. □

#### 4.2.3. AWSS AR Sources

**Definition** **6** **(AR process).** *A real zero-mean random process ${\left\{x_{n}\right\}}_{n\in N}$ is said to be AR if*

$$x_{n}=w_{n}-\sum_{j=1}^{n-1}a_{-j}x_{n-j}\;\forall n\in N,$$

*where $a_{-j}\in R$ for all j∈N, and ${\left\{w_{n}\right\}}_{n\in N}$ is a real zero-mean random process satisfying that $E\left(w_{j}w_{k}\right)=\delta_{j,k}\sigma_{w}^{2}$ for all j,k∈N with $\sigma_{w}^{2}>0$. If there exists p∈N such that $a_{-j}=0$ for all j>p, then ${\left\{x_{n}\right\}}_{n\in N}$ is called an AR(p) process.*

**Theorem** **5.** *Suppose that ${\left\{x_{n}\right\}}_{n\in N}$ is an AR(p) process as in Definition 6. Let $a\left(\omega \right)=1+\sum_{j=1}^{p}a_{-j}e^{-j\omega i}$ for all ω∈R. Assume that a(ω)≠0 for all ω∈R, and that there exists K>0 such that ${∥{\left(T_{n}\left(a\right)\right)}^{-1}∥}_{2}\leq K$ for all n∈N. Then, ${\left\{x_{n}\right\}}_{n\in N}$ is AWSS with PSD $\frac{\sigma_{w}^{2}}{{\left|a\right|}^{2}}$, and*

$${\tilde{P}}_{n}\left(D\right)-P_{n}\left(D\right)=O\left(1/\sqrt{n}\right).$$

*for all $D\in \left(0,\underset{n\in N}{\inf}\lambda_{n}\left(E(x_{n:1}x_{n:1}^{⊤})\right)\right]$.*

**Proof.**  It is a direct consequence of Theorem 3. □

#### 4.2.4. WSS Sources

**Theorem** **6.** *Suppose that ${\left\{x_{n}\right\}}_{n\in N}$ is a WSS process as in Definition 2 with PSD f. Assume that f(ω)≠0 for all ω∈R and that there exists m∈N such that $f_{k}=0$ whenever |k|>m. Then,*

$${\tilde{P}}_{n}\left(D\right)-P_{n}\left(D\right)=O\left(1/\sqrt{n}\right).$$

*for all $D\in \left(0,\min(f)\right]$.*

**Proof.**  See Appendix F. □

## 5. Numerical Example

**Figure 5:**
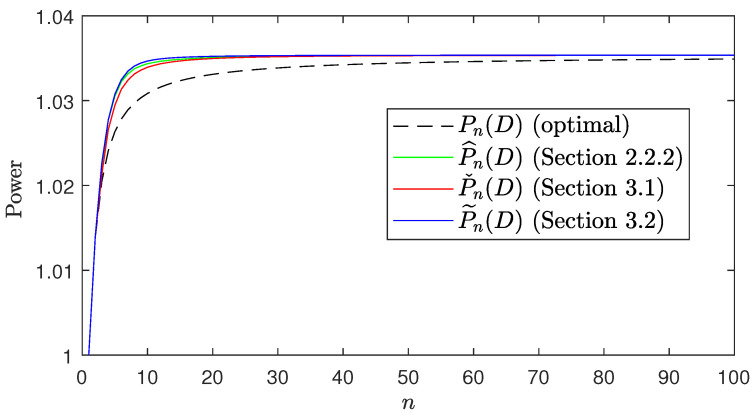
Theoretical average transmission powers of the considered schemes.

**Figure 6:**
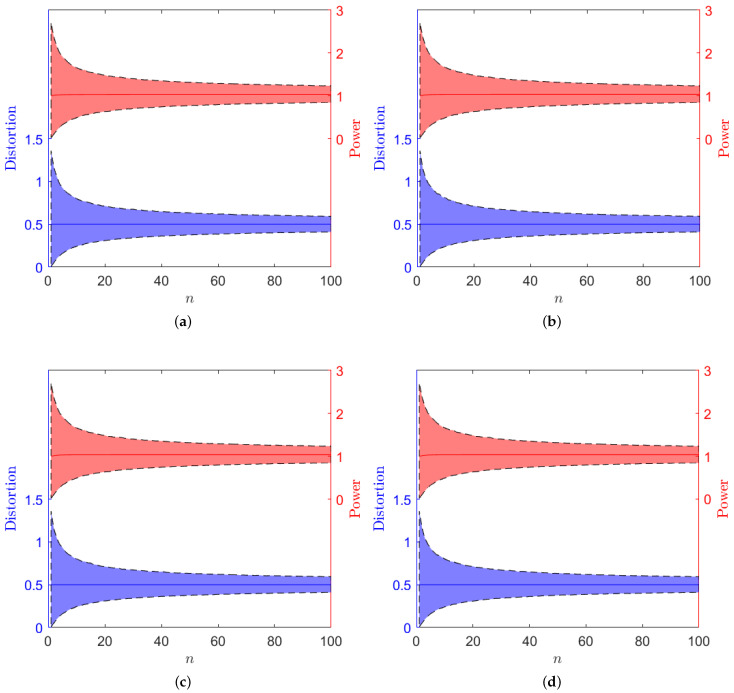
Interval between the 10th and the 90th percentile for the actual transmission power and the actual distortion (shaded) and theoretical average transmission power and distortion (solid line) in each of the considered schemes. (**a**) Optimal linear coding scheme (Section 2.2.1). (**b**) Low-power alternative (Section 3.1). (**c**) DFT-based alternative (Section 2.2.2). (**d**) DFT/IDFT alternative (Section 3.2).

In this section we give a numerical example. We consider an ARMA(1,1) process ${\left\{x_{n}\right\}}_{n\in N}$ with $a_{-1}=-\frac{1}{2}$, $b_{-1}=-\frac{1}{3}$ and $\sigma_{w}^{2}=1$, channel noise variance $\sigma^{2}=1$ and an average distortion constraint D=0.5. Figure 5 shows the theoretical value of the average transmission power required for each of the considered schemes with n∈{1,…,100}. It can be observed how the graphs of the average transmission power of the different schemes follow the inequalities in (9), and how the transmission power of the different schemes get closer as *n* increases. Moreover, we have simulated the transmission of 20,000 samples of the considered ARMA(1,1) process for n∈{1,…,100}. Figure 6 shows the 10th and the 90th percentile of the power and distortion of the samples.

## 6. Conclusions

In this paper, two new low-complexity linear coding schemes for transmitting *n*-dimensional vectors by using *n* times an AWGN channel have been presented. These schemes are based on the DFT. The performance of these schemes, along with another DFT-based scheme that had been previously presented, has been analyzed in comparison with the optimal scheme among the linear ones. These three DFT-based schemes allow good performance in terms of distortion using low-cost, low-power hardware.

In particular, it has been proved that, under a maximum average distortion constraint, the considered low-complexity schemes require the same average transmission power as the optimal linear scheme for AWSS sources when the block length, *n*, tends to infinity. Moreover, it has been proved that for certain types of AWSS sources (namely WSS, MA, AWSS AR and AWSS ARMA), the difference between the transmission power of each of the three alternative schemes and the transmission power of the optimal linear scheme decreases as $O\left(1/\sqrt{n}\right)$. Therefore, their performance will be similar to that of the optimal linear coding scheme even for small values of *n*. In other words, replacing the optimal linear scheme with any of the schemes studied here will not have, even for small block sizes, a large penalty in terms of transmission power, while it will lead to a noticeable reduction in complexity. 

## Data Availability

Not applicable.

## Abbreviations

The following abbreviations are used in this manuscript:

ADC	Analog-to-digital converter
AR	Autoregressive
ARMA	Autoregressive moving average
AWGN	Additive white Gaussian noise
AWSS	Asymptotically wide-sense stationary
DFT	Discrete Fourier transform
FFT	Fast Fourier transform
IDFT	Inverse discrete Fourier transform
iid	Independent and identically distributed
IoT	Internet of Things
MA	Moving average
WSS	Wide-sense stationary

## Appendix A. Average Transmission Power and Average Distortion of the Low-Power Alternative

We first recall the basic symmetry property of the DFT.

**Lemma** **A1.**
*Let $x_{j},y_{j}\in C$ for all j∈{1,…,n}, with n∈N. If $y_{n:1}$ is the DFT of $x_{n:1}$, i.e., $y_{n:1}=V_{n}^{*}x_{n:1}$, the two following assertions are equivalent:*

1. $x_{n:1}\in R^{n\times 1}$.
2. *$y_{j}=\bar{y_{n-j}}$ for all j∈{1,…,n−1} and $y_{n}\in R$.*

Now we show that ${\overset{ˇ}{\alpha }}_{j}$ and ${\overset{ˇ}{\beta }}_{j}$ in (3) and (4), respectively, are well defined. If $D\in \left(0,\lambda_{n}\left(E\left(x_{n:1}x_{n:1}^{⊤}\right)\right)\right]$, applying Theorems 1 and 2 from [9], $E\left(z_{j}^{2}\right)\geq \lambda_{n}\left(E\left(x_{n:1}x_{n:1}^{⊤}\right)\right)\geq D>0$ for all j∈{1,…,n}. Hence ${\left(\frac{1}{n}\sum_{j=1}^{n}\sqrt{E\left(z_{j}^{2}\right)}\right)}^{2}\geq {\left(\frac{1}{n}\sum_{j=1}^{n}\sqrt{D}\right)}^{2}=D$ and, consequently, ${\overset{ˇ}{P}}_{n}\left(D\right)\geq 0$. Thus,

$$\sqrt{\frac{\sigma^{2}\left(\sigma^{2}+{\overset{ˇ}{P}}_{n}\left(D\right)\right)E\left(z_{j}^{2}\right)}{D}}\geq \sigma^{2}\sqrt{\frac{E\left(z_{j}^{2}\right)}{D}}\geq \sigma^{2}$$

and, therefore, ${\overset{ˇ}{\alpha }}_{j}$ is well defined in (3). Furthermore, ${\overset{ˇ}{\alpha }}_{j}^{2}E\left(z_{j}^{2}\right)+\sigma^{2}>0$ and, consequently, ${\overset{ˇ}{\beta }}_{j}$ is well defined in (4).

Next, we show that the average transmission power of the coding scheme shown in Figure 3 is ${\overset{ˇ}{P}}_{n}\left(D\right)$ given in (5):

$$\begin{array}{cc} \; & \frac{1}{n}E\left({∥u_{n:1}∥}_{F}^{2}\right)=\frac{1}{n}E\left(\sum_{j=1}^{n}u_{j}^{2}\right)=\frac{1}{n}\sum_{j=1}^{n}E\left({\overset{ˇ}{\alpha }}_{j}^{2}z_{j}^{2}\right)=\frac{1}{n}\sum_{j=1}^{n}{\overset{ˇ}{\alpha }}_{j}^{2}E\left(z_{j}^{2}\right)\\ & =\frac{1}{n}\sum_{j=1}^{n}\left(\sqrt{\frac{\sigma^{2}\left(\sigma^{2}+{\overset{ˇ}{P}}_{n}\left(D\right)\right)E\left(z_{j}^{2}\right)}{D}}-\sigma^{2}\right)=\frac{\sigma^{2}}{D}\left(\sqrt{\frac{D}{\sigma^{2}}}\sqrt{\sigma^{2}+{\overset{ˇ}{P}}_{n}\left(D\right)}\frac{1}{n}\sum_{j=1}^{n}\sqrt{E\left(z_{j}^{2}\right)}-D\right)\\ \; & =\frac{\sigma^{2}}{D}\left(\sqrt{\frac{D}{\sigma^{2}}}\sqrt{\sigma^{2}+\frac{\sigma^{2}}{D}\left({\left(\frac{1}{n}\sum_{j=1}^{n}\sqrt{E\left(z_{j}^{2}\right)}\right)}^{2}-D\right)}\frac{1}{n}\sum_{j=1}^{n}\sqrt{E\left(z_{j}^{2}\right)}-D\right)\\ \; & =\frac{\sigma^{2}}{D}\left({\left(\frac{1}{n}\sum_{j=1}^{n}\sqrt{E\left(z_{j}^{2}\right)}\right)}^{2}-D\right)={\overset{ˇ}{P}}_{n}\left(D\right). \end{array}$$

Finally, we show that $\frac{1}{n}E\left({∥x_{n:1}-\tilde{x_{n:1}}∥}_{F}^{2}\right)=D$. From Figure 3, observe that $\tilde{y_{j}}=\bar{\tilde{y_{n-j}}}$ for $j\in \left\{1,\ldots ,\left\lfloor \frac{n-1}{2}\right\rfloor \right\}$, where ⌊a⌋ represents the largest integer lower than or equal to a∈R. Furthermore, as $E\left(x_{j}\nu_{k}\right)=0$ for all j,k∈{1,…,n} and given that each $z_{j}$ with j∈{1,…,n} is a linear combination of $x_{1},\ldots ,x_{n}$, $E\left(z_{j}\nu_{k}\right)=0$ for all j,k∈{1,…,n}. Consequently, since the Frobenius norm is unitarily invariant, we have:

1nExn:1−xn:1˜F2=1nEVn*xn:1−xn:1˜F2=1nEyn:1−yn:1˜F2=1nE∑j∈{n2,n}∩Nyj−yj˜2+∑k=1n−12yk−yk˜2+∑j=n+12n−1yj−yj˜2=1nE∑j∈{n2,n}∩Nyj−yj˜2+∑k=1n−12yn−k¯−yn−k˜¯2+∑j=n+12n−1yj−yj˜2=1nE∑j∈{n2,n}∩Nyj−yj˜2+∑j=n+12n−1yj¯−yj˜¯2+∑j=n+12n−1yj−yj˜2=1nE∑j∈{n2,n}∩Nyj−yj˜2+∑j=n+12n−1yj−yj˜¯2+∑j=n+12n−1yj−yj˜2=1nE∑j∈{n2,n}∩Nyj−yj˜2+2∑j=n+12n−1yj−yj˜2=1nE∑j∈{n2,n}∩Nyj−yj˜2+2∑j=n+12n−1Reyj−yj˜2+Imyj−yj˜2=1nE∑j∈{n2,n}∩Nyj−yj˜2+2∑j=n+12n−1yj−yj˜^F2=1nE∑j∈{n2,n}∩Nyj−yj˜2+2∑j=n+12n−1Uyj^⊤yj−yj˜^F2=1nE∑j∈{n2,n}∩Nyj−yj˜2+∑j=n+12n−12Uyj^⊤yj^−2Uyj^⊤yj˜^F2=1nE∑j=1nzj−zj˜2=1nE∑j=1nzj−βˇjαˇjzj+νj2=1n∑j=1nE1−βˇjαˇjzj−βˇjνj2=1n∑j=1n1−βˇjαˇj2Ezj2+βˇj2Eνj2=1n∑j=1n1−αˇj2Ezj2αˇj2Ezj2+σ22Ezj2+αˇjEzj2αˇj2Ezj2+σ22σ2=1n∑j=1nσ22αˇj2Ezj2+σ22Ezj2+αˇjEzj22αˇj2Ezj2+σ22σ2=1n∑j=1nEzj2σ2αˇj2Ezj2+σ2αˇj2Ezj2+σ22=1n∑j=1nEzj2σ21E(zj2)σ2(σ2+Pˇn(D))E(zj2)D−σ2Ezj2+σ2=σ2Dσ2+Pˇn(D)1n∑j=1nEzj2=σ2Dσ2+σ2D1n∑j=1nEzj22−D1n∑j=1nEzj2=D1n∑j=1nEzj2D+1n∑j=1nEzj22−D=D.

## Appendix B. Average Transmission Power and Average Distortion of the DFT/IDFT Alternative

We first show that ${\tilde{\alpha }}_{j}$ and ${\tilde{\beta }}_{j}$ in (6) and (7), respectively, are well defined. If $D\in \left(0,\lambda_{n}\left(E\left(x_{n:1}x_{n:1}^{⊤}\right)\right)\right]$, applying Theorem 1 from [9], $E\left({\left|y_{j}\right|}^{2}\right)\geq \lambda_{n}\left(E\left(x_{n:1}x_{n:1}^{⊤}\right)\right)\geq D>0$ for all j∈{1,…,n}. Hence ${\left(\frac{1}{n}\sum_{j=1}^{n}\sqrt{E\left({\left|y_{j}\right|}^{2}\right)}\right)}^{2}\geq {\left(\frac{1}{n}\sum_{j=1}^{n}\sqrt{D}\right)}^{2}=D$ and, consequently, ${\tilde{P}}_{n}\left(D\right)\geq 0$. Thus,

$$\sqrt{\frac{\sigma^{2}\left(\sigma^{2}+{\tilde{P}}_{n}\left(D\right)\right)E\left({\left|y_{j}\right|}^{2}\right)}{D}}\geq \sigma^{2}\sqrt{\frac{E\left({\left|y_{j}\right|}^{2}\right)}{D}}\geq \sigma^{2}$$

and, therefore, ${\tilde{\alpha }}_{j}$ is well defined in (6). Furthermore, ${\tilde{\alpha }}_{j}^{2}E\left({\left|y_{j}\right|}^{2}\right)+\sigma^{2}>0$ and, consequently, ${\tilde{\beta }}_{j}$ is well defined in (7).

We now show that the output of the encoder, $u_{n:1}=V_{n}\mathrm{diag}\left({\tilde{\alpha }}_{n},\ldots ,{\tilde{\alpha }}_{1}\right)y_{n:1}$, is real. Observe that $u_{n:1}$ is real if and only if $V_{n}^{*}u_{n:1}=\mathrm{diag}\left({\tilde{\alpha }}_{n},\ldots ,{\tilde{\alpha }}_{1}\right)y_{n:1}$ fulfills the conditions given in assertion 2 of Lemma A1, i.e., ${\tilde{\alpha }}_{n}y_{n}\in R$ and ${\tilde{\alpha }}_{j}y_{j}=\bar{{\tilde{\alpha }}_{n-j}y_{n-j}}$ for j∈{1,…,n−1}. From (6), ${\tilde{\alpha }}_{j}$ with j∈{1,…,n} are always real numbers. Since $y_{n:1}$ is the DFT of the real vector $x_{n:1}$, $y_{n}$ is real, and so is ${\tilde{\alpha }}_{n}y_{n}$. Furthermore, given that $y_{j}=\bar{y_{n-j}}$ for j∈{1,…,n−1}, $E\left({\left|y_{j}\right|}^{2}\right)=E\left({\left|y_{n-j}\right|}^{2}\right)$ for j∈{1,…,n−1}. Then, from (6), ${\tilde{\alpha }}_{j}={\tilde{\alpha }}_{n-j}$ for j∈{1,…,n−1}, and $\bar{{\tilde{\alpha }}_{n-j}y_{n-j}}={\tilde{\alpha }}_{n-j}\bar{y_{n-j}}={\tilde{\alpha }}_{j}y_{j}$ for j∈{1,…,n−1}.

Next, we show that the average transmission power of the coding scheme shown in Figure 4 is ${\tilde{P}}_{n}\left(D\right)$ given in (8). Since the Frobenius norm is unitarily invariant, we have:

$$\begin{array}{cc} \; & \frac{1}{n}E\left({∥u_{n:1}∥}_{F}^{2}\right)=\frac{1}{n}E\left({∥V_{n}\mathrm{diag}\left({\tilde{\alpha }}_{n},\ldots ,{\tilde{\alpha }}_{1}\right)V_{n}^{*}x_{n:1}∥}_{F}^{2}\right)=\frac{1}{n}E\left({∥\mathrm{diag}\left({\tilde{\alpha }}_{n},\ldots ,{\tilde{\alpha }}_{1}\right)V_{n}^{*}x_{n:1}∥}_{F}^{2}\right)\\ \; & =\frac{1}{n}\sum_{j=1}^{n}{\tilde{\alpha }}_{j}^{2}E\left({\left|y_{j}\right|}^{2}\right)=\frac{1}{n}\sum_{j=1}^{n}\left(\sqrt{\frac{\sigma^{2}\left(\sigma^{2}+{\tilde{P}}_{n}\left(D\right)\right)E\left({\left|y_{j}\right|}^{2}\right)}{D}}-\sigma^{2}\right)\\ \; & =\frac{\sigma^{2}}{D}\left(\sqrt{\frac{D}{\sigma^{2}}}\sqrt{\sigma^{2}+{\tilde{P}}_{n}\left(D\right)}\frac{1}{n}\sum_{j=1}^{n}\sqrt{E\left({\left|y_{j}\right|}^{2}\right)}-D\right)\\ \; & =\frac{\sigma^{2}}{D}\left(\sqrt{\frac{D}{\sigma^{2}}}\sqrt{\sigma^{2}+\frac{\sigma^{2}}{D}\left({\left(\frac{1}{n}\sum_{j=1}^{n}\sqrt{E\left({\left|y_{j}\right|}^{2}\right)}\right)}^{2}-D\right)}\frac{1}{n}\sum_{j=1}^{n}\sqrt{E\left({\left|y_{j}\right|}^{2}\right)}-D\right)\\ \; & =\frac{\sigma^{2}}{D}\left({\left(\frac{1}{n}\sum_{j=1}^{n}\sqrt{E\left({\left|y_{j}\right|}^{2}\right)}\right)}^{2}-D\right)={\tilde{P}}_{n}\left(D\right). \end{array}$$

Finally, we show that $\frac{1}{n}E\left({∥x_{n:1}-\tilde{x_{n:1}}∥}_{F}^{2}\right)=D$. As $E\left(x_{j}\nu_{k}\right)=0$ for all j,k∈{1,…,n} then $E\left(y_{n:1}\nu_{n:1}^{*}\right)=E\left(V_{n}^{*}x_{n:1}\nu_{n:1}^{*}\right)=V_{n}^{*}E\left(x_{n:1}\nu_{n:1}^{*}\right)=0_{n\times n}$, where $0_{n\times n}$ is the n×n zero matrix. Consequently, we have:

$$\begin{array}{cc} \; & \frac{1}{n}E\left({∥x_{n:1}-\tilde{x_{n:1}}∥}_{F}^{2}\right)=\frac{1}{n}E\left({∥V_{n}^{*}\left(x_{n:1}-\tilde{x_{n:1}}\right)∥}_{F}^{2}\right)=\frac{1}{n}E\left({∥y_{n:1}-\tilde{y_{n:1}}∥}_{F}^{2}\right)\\ \; & =\frac{1}{n}E\left({∥y_{n:1}-\mathrm{diag}\left({\tilde{\beta }}_{n},\ldots ,{\tilde{\beta }}_{1}\right)V_{n}^{*}\left(V_{n}\mathrm{diag}\left({\tilde{\alpha }}_{n},\ldots ,{\tilde{\alpha }}_{1}\right)y_{n:1}+\nu_{n:1}\right)∥}_{F}^{2}\right)\\ \; & =\frac{1}{n}E\left({∥y_{n:1}-\mathrm{diag}\left({\tilde{\beta }}_{n},\ldots ,{\tilde{\beta }}_{1}\right)\mathrm{diag}\left({\tilde{\alpha }}_{n},\ldots ,{\tilde{\alpha }}_{1}\right)y_{n:1}-\mathrm{diag}\left({\tilde{\beta }}_{n},\ldots ,{\tilde{\beta }}_{1}\right)V_{n}^{*}\nu_{n:1}∥}_{F}^{2}\right)\\ \; & =\frac{1}{n}E\left({∥\mathrm{diag}\left(1-{\tilde{\beta }}_{n}{\tilde{\alpha }}_{n},\ldots ,1-{\tilde{\beta }}_{1}{\tilde{\alpha }}_{1}\right)y_{n:1}-\mathrm{diag}\left({\tilde{\beta }}_{n},\ldots ,{\tilde{\beta }}_{1}\right)V_{n}^{*}\nu_{n:1}∥}_{F}^{2}\right)\\ \; & \begin{array}{c} =\frac{1}{n}E\left(\mathrm{tr}\left(\left(\mathrm{diag}\left(1-{\tilde{\beta }}_{n}{\tilde{\alpha }}_{n},\ldots ,1-{\tilde{\beta }}_{1}{\tilde{\alpha }}_{1}\right)y_{n:1}-\mathrm{diag}\left({\tilde{\beta }}_{n},\ldots ,{\tilde{\beta }}_{1}\right)V_{n}^{*}\nu_{n:1}\right)\right.\right.\\ \left.\left.\cdot {\left(\mathrm{diag}\left(1-{\tilde{\beta }}_{n}{\tilde{\alpha }}_{n},\ldots ,1-{\tilde{\beta }}_{1}{\tilde{\alpha }}_{1}\right)y_{n:1}-\mathrm{diag}\left({\tilde{\beta }}_{n},\ldots ,{\tilde{\beta }}_{1}\right)V_{n}^{*}\nu_{n:1}\right)}^{*}\right)\right) \end{array}\\ \; & \begin{array}{c} =\frac{1}{n}\mathrm{tr}\left(E\left(\left(\mathrm{diag}\left(1-{\tilde{\beta }}_{n}{\tilde{\alpha }}_{n},\ldots ,1-{\tilde{\beta }}_{1}{\tilde{\alpha }}_{1}\right)y_{n:1}-\mathrm{diag}\left({\tilde{\beta }}_{n},\ldots ,{\tilde{\beta }}_{1}\right)V_{n}^{*}\nu_{n:1}\right)\right.\right.\\ \left.\left.\cdot {\left(\mathrm{diag}\left(1-{\tilde{\beta }}_{n}{\tilde{\alpha }}_{n},\ldots ,1-{\tilde{\beta }}_{1}{\tilde{\alpha }}_{1}\right)y_{n:1}-\mathrm{diag}\left({\tilde{\beta }}_{n},\ldots ,{\tilde{\beta }}_{1}\right)V_{n}^{*}\nu_{n:1}\right)}^{*}\right)\right) \end{array}\\ \; & \begin{array}{c} =\frac{1}{n}\mathrm{tr}\left(\mathrm{diag}\left(1-{\tilde{\beta }}_{n}{\tilde{\alpha }}_{n},\ldots ,1-{\tilde{\beta }}_{1}{\tilde{\alpha }}_{1}\right)E\left(y_{n:1}y_{n:1}^{*}\right)\mathrm{diag}\left(1-{\tilde{\beta }}_{n}{\tilde{\alpha }}_{n},\ldots ,1-{\tilde{\beta }}_{1}{\tilde{\alpha }}_{1}\right)\right.\\ \left.+\mathrm{diag}\left({\tilde{\beta }}_{n},\ldots ,{\tilde{\beta }}_{1}\right)V_{n}^{*}E\left(\nu_{n:1}\nu_{n:1}^{⊤}\right)V_{n}\mathrm{diag}\left({\tilde{\beta }}_{n},\ldots ,{\tilde{\beta }}_{1}\right)\right) \end{array}\\ \; & \begin{array}{c} =\frac{1}{n}\mathrm{tr}\left(\mathrm{diag}\left(1-{\tilde{\beta }}_{n}{\tilde{\alpha }}_{n},\ldots ,1-{\tilde{\beta }}_{1}{\tilde{\alpha }}_{1}\right)E\left(y_{n:1}y_{n:1}^{*}\right)\mathrm{diag}\left(1-{\tilde{\beta }}_{n}{\tilde{\alpha }}_{n},\ldots ,1-{\tilde{\beta }}_{1}{\tilde{\alpha }}_{1}\right)\right.\\ \left.+\mathrm{diag}\left({\tilde{\beta }}_{n},\ldots ,{\tilde{\beta }}_{1}\right)V_{n}^{*}\sigma^{2}I_{n}V_{n}\mathrm{diag}\left({\tilde{\beta }}_{n},\ldots ,{\tilde{\beta }}_{1}\right)\right) \end{array}\\ \; & =\frac{1}{n}\sum_{j=1}^{n}\left({\left(1-{\tilde{\beta }}_{j}{\tilde{\alpha }}_{j}\right)}^{2}E\left({\left|y_{j}\right|}^{2}\right)+{\tilde{\beta }}_{j}^{2}\sigma^{2}\right)\\ \; & =\frac{1}{n}\sum_{j=1}^{n}\left({\left(1-\frac{{\tilde{\alpha }}_{j}^{2}E\left({\left|y_{j}\right|}^{2}\right)}{{\tilde{\alpha }}_{j}^{2}E\left({\left|y_{j}\right|}^{2}\right)+\sigma^{2}}\right)}^{2}E\left({\left|y_{j}\right|}^{2}\right)+{\left(\frac{{\tilde{\alpha }}_{j}E\left({\left|y_{j}\right|}^{2}\right)}{{\tilde{\alpha }}_{j}^{2}E\left({\left|y_{j}\right|}^{2}\right)+\sigma^{2}}\right)}^{2}\sigma^{2}\right)\\ \; & =\frac{1}{n}\sum_{j=1}^{n}\left(\frac{{\left(\sigma^{2}\right)}^{2}}{{\left({\tilde{\alpha }}_{j}^{2}E\left({\left|y_{j}\right|}^{2}\right)+\sigma^{2}\right)}^{2}}E\left({\left|y_{j}\right|}^{2}\right)+\frac{{\left({\tilde{\alpha }}_{j}E\left({\left|y_{j}\right|}^{2}\right)\right)}^{2}}{{\left({\tilde{\alpha }}_{j}^{2}E\left({\left|y_{j}\right|}^{2}\right)+\sigma^{2}\right)}^{2}}\sigma^{2}\right)\\ \; & =\frac{1}{n}\sum_{j=1}^{n}E\left({\left|y_{j}\right|}^{2}\right)\sigma^{2}\frac{{\tilde{\alpha }}_{j}^{2}E\left({\left|y_{j}\right|}^{2}\right)+\sigma^{2}}{{\left({\tilde{\alpha }}_{j}^{2}E\left({\left|y_{j}\right|}^{2}\right)+\sigma^{2}\right)}^{2}}\\ \; & =\frac{1}{n}\sum_{j=1}^{n}\frac{E\left({\left|y_{j}\right|}^{2}\right)\sigma^{2}}{\frac{1}{E({\left|y_{j}\right|}^{2})}\left(\sqrt{\frac{\sigma^{2}\left(\sigma^{2}+{\tilde{P}}_{n}\left(D\right)\right)E\left({\left|y_{j}\right|}^{2}\right)}{D}}-\sigma^{2}\right)E\left({\left|y_{j}\right|}^{2}\right)+\sigma^{2}}\\ \; & =\frac{\sqrt{\sigma^{2}D}}{\sqrt{\sigma^{2}+{\tilde{P}}_{n}\left(D\right)}}\frac{1}{n}\sum_{j=1}^{n}\sqrt{E\left({\left|y_{j}\right|}^{2}\right)}\\ \; & =\frac{\sqrt{\sigma^{2}D}}{\sqrt{\sigma^{2}+\frac{\sigma^{2}}{D}\left({\left(\frac{1}{n}\sum_{j=1}^{n}\sqrt{E\left({\left|y_{j}\right|}^{2}\right)}\right)}^{2}-D\right)}}\frac{1}{n}\sum_{j=1}^{n}\sqrt{E\left({\left|y_{j}\right|}^{2}\right)}\\ \; & =D\frac{\frac{1}{n}\sum_{j=1}^{n}\sqrt{E\left({\left|y_{j}\right|}^{2}\right)}}{\sqrt{D+{\left(\frac{1}{n}\sum_{j=1}^{n}\sqrt{E\left({\left|y_{j}\right|}^{2}\right)}\right)}^{2}-D}}=D, \end{array}$$

where tr(·) denotes the trace of a matrix.

## Appendix C. Proof of Theorem 1

We first need to introduce the following result.

**Lemma** **A2.**
*Let $A\in R^{2\times 2}$ be a symmetric positive semi-definite matrix. Then,*

$$\sqrt{\lambda_{1}\left(A\right)}+\sqrt{\lambda_{2}\left(A\right)}\leq \sqrt{{\left[A\right]}_{1,1}}+\sqrt{{\left[A\right]}_{2,2}}.$$

**Proof.** 

$$\begin{array}{cc} \; & \sqrt{\lambda_{1}\left(A\right)}+\sqrt{\lambda_{2}\left(A\right)}=\sqrt{{\left(\sqrt{\lambda_{1}\left(A\right)}+\sqrt{\lambda_{2}\left(A\right)}\right)}^{2}}=\sqrt{\lambda_{1}\left(A\right)+\lambda_{2}\left(A\right)+2\sqrt{\lambda_{1}\left(A\right)\lambda_{2}\left(A\right)}}\\ \; & =\sqrt{\mathrm{tr}\left(A\right)+2\sqrt{\det(A)}}=\sqrt{{\left[A\right]}_{1,1}+{\left[A\right]}_{2,2}+2\sqrt{{\left[A\right]}_{1,1}{\left[A\right]}_{2,2}-{\left[A\right]}_{1,2}^{2}}}\\ \; & \leq \sqrt{{\left[A\right]}_{1,1}+{\left[A\right]}_{2,2}+2\sqrt{{\left[A\right]}_{1,1}{\left[A\right]}_{2,2}}}=\sqrt{{\left(\sqrt{{\left[A\right]}_{1,1}}+\sqrt{{\left[A\right]}_{2,2}}\right)}^{2}}=\sqrt{{\left[A\right]}_{1,1}}+\sqrt{{\left[A\right]}_{2,2}}. \end{array}$$

*□*

Next, we prove Theorem 1.

**Proof.** $P_{n}\left(D\right)\leq {\overset{ˇ}{P}}_{n}\left(D\right)$ holds because of the optimality of the scheme presented in [4]. We now show that ${\overset{ˇ}{P}}_{n}\left(D\right)\leq {\hat{P}}_{n}\left(D\right)$. Applying Lemmas A1 and A2 yields

∑j∈{n2,n}∩NEyj2+∑j=n+12n−12EReyj2+∑j=1n−122EImyj2−∑j∈{n2,n}∩NEyj2−∑j=n+12n−12λ1Eyj^yj^⊤+2λ2Eyj^yj^⊤=∑j=n+12n−12EReyj2+∑j=1n−122E−Imyn−j2−∑j=n+12n−12λ1Eyj^yj^⊤+2λ2Eyj^yj^⊤=∑j=n+12n−12EReyj2+∑j=n+12n−12EImyj2−∑j=n+12n−12λ1Eyj^yj^⊤+2λ2Eyj^yj^⊤=∑j=n+12n−1EReyj2+EImyj2−λ1Eyj^yj^⊤−λ2Eyj^yj^⊤≥0.

Hence, from (2) and (5), ${\overset{ˇ}{P}}_{n}\left(D\right)\leq {\hat{P}}_{n}\left(D\right)$ holds. Finally, we show that ${\hat{P}}_{n}\left(D\right)\leq {\tilde{P}}_{n}\left(D\right)$. Reasoning as in Step 6 of the proof of ([5] Theorem 1) we have:

∑j=1nEyj2≥∑j∈{n2,n}∩NEyj2+∑j=n+12n−12EReyj2+∑j=1n−122EImyj2.

Hence, from (2) and (8), ${\hat{P}}_{n}\left(D\right)\leq {\tilde{P}}_{n}\left(D\right)$ holds. □

## Appendix D. Proof of Theorem 2

**Proof.** We first prove that $\lim_{n\to \infty }P_{n}\left(D\right)=\frac{\sigma^{2}}{D}\left({\left(\frac{1}{2\pi }\int_{0}^{2\pi }\sqrt{f(\omega )}d\omega \right)}^{2}-D\right)$. From (1) and ([10] Theorem 6.6),

$$\begin{array}{cc} \lim_{n\to \infty }P_{n}\left(D\right) & =\lim_{n\to \infty }\frac{\sigma^{2}}{D}\left({\left(\frac{1}{n}\sum_{j=1}^{n}\sqrt{\lambda_{j}\left(E\left(x_{n:1}x_{n:1}^{⊤}\right)\right)}\right)}^{2}-D\right)\\ \; & =\frac{\sigma^{2}}{D}\left({\left(\lim_{n\to \infty }\frac{1}{n}\sum_{j=1}^{n}\sqrt{\lambda_{j}\left(E\left(x_{n:1}x_{n:1}^{⊤}\right)\right)}\right)}^{2}-D\right)\\ \; & =\frac{\sigma^{2}}{D}\left({\left(\frac{1}{2\pi }\int_{0}^{2\pi }\sqrt{f(\omega )}d\omega \right)}^{2}-D\right). \end{array}$$

To prove the remaining equalities, from Theorem 1, we have to show that

$$\lim_{n\to \infty }\left({\tilde{P}}_{n}\left(D\right)-P_{n}\left(D\right)\right)=0.$$

To that end, we need to introduce some notation. If $A_{n}$ is an n×n matrix, $C_{A_{n}}$ is the n×n circulant matrix

$$C_{A_{n}}=V_{n}\mathrm{diag}\left({\left[V_{n}^{*}A_{n}V_{n}\right]}_{1,1},\ldots ,{\left[V_{n}^{*}A_{n}V_{n}\right]}_{n,n}\right)V_{n}^{*}.$$

Moreover, we denote by $C_{n}\left(f\right)$ the n×n circulant matrix defined as

$$C_{n}\left(f\right)=V_{n}\mathrm{diag}\left(f\left(0\right),f\left(\frac{2\pi }{n}\right),\ldots ,f\left(\frac{2\pi (n-1)}{n}\right)\right)V_{n}^{*}.$$

Due to the optimality of the scheme proposed in [4], and from the proof of assertion 4 of Lemma 1 in [6],

(A1) $$\begin{array}{cc} \; & 0\leq {\tilde{P}}_{n}\left(D\right)-P_{n}\left(D\right)\\ \; & =\frac{\sigma^{2}}{D}\left({\left(\frac{1}{n}\sum_{j=1}^{n}\sqrt{E\left({\left|y_{j}\right|}^{2}\right)}\right)}^{2}-{\left(\frac{1}{n}\sum_{j=1}^{n}\sqrt{\lambda_{j}\left(E\left(x_{n:1}x_{n:1}^{⊤}\right)\right)}\right)}^{2}\right)\\ \; & \leq \frac{\sigma^{2}}{D}\sqrt{\frac{\lambda_{1}\left(E\left(x_{n:1}x_{n:1}^{⊤}\right)\right)}{\lambda_{n}\left(E\left(x_{n:1}x_{n:1}^{⊤}\right)\right)}}\frac{{∥E\left(x_{n:1}x_{n:1}^{⊤}\right)-C_{x_{n:1}}∥}_{F}}{\sqrt{n}}\\ \; & \leq \frac{\sigma^{2}}{D}\sqrt{\frac{{∥E\left(x_{n:1}x_{n:1}^{⊤}\right)∥}_{2}}{\underset{m\in N}{\inf}\lambda_{m}\left(E\left(x_{m:1}x_{m:1}^{⊤}\right)\right)}}\frac{{∥E\left(x_{n:1}x_{n:1}^{⊤}\right)-C_{x_{n:1}}∥}_{F}}{\sqrt{n}} \end{array}$$

where $C_{x_{n:1}}=C_{E\left(x_{n:1}x_{n:1}^{⊤}\right)}$. Observe that, from Definition 3, $\left\{{∥E\left(x_{n:1}x_{n:1}^{⊤}\right)∥}_{2}\right\}$ is bounded. Hence, to finish the proof we only have to show that

(A2) $$\lim_{n\to \infty }\frac{{∥E\left(x_{n:1}x_{n:1}^{⊤}\right)-C_{x_{n:1}}∥}_{F}}{\sqrt{n}}=0.$$

By combining Equation (16) in [11], Definition 3 and Lemma 6.1 in [10], (A2) holds. □

## Appendix E. Proof of Theorem 3

**Proof.** Since ${∥{\left(T_{n}\left(a\right)\right)}^{-1}∥}_{2}\leq K_{1}$ for all n∈N, $\sigma_{n}\left(T_{n}\left(a\right)\right)\geq \frac{1}{K_{1}}>0$ for every n∈N. As a(ω)≠0 for all ω∈R, from Theorem 8 in [12], ${\left\{x_{n}\right\}}_{n\in N}$ is AWSS with PSD $\sigma_{w}^{2}\frac{{\left|b\right|}^{2}}{{\left|a\right|}^{2}}$. Next, we show that $\underset{n\in N}{\inf}\lambda_{n}\left(E\left(x_{n:1}x_{n:1}^{⊤}\right)\right)>0$. Observe that (10) can be rewritten as

$$T_{n}\left(a\right)x_{n:1}=T_{n}\left(b\right)w_{n:1}\;\forall n\in N.$$

Then,

$$E\left(x_{n:1}x_{n:1}^{⊤}\right)=\sigma_{w}^{2}{\left(T_{n}\left(a\right)\right)}^{-1}T_{n}\left(b\right){\left(T_{n}\left(b\right)\right)}^{⊤}{\left({\left(T_{n}\left(a\right)\right)}^{-1}\right)}^{⊤}$$

and

$${\left(E\left(x_{n:1}x_{n:1}^{⊤}\right)\right)}^{-1}=\frac{1}{\sigma_{w}^{2}}{\left(T_{n}\left(a\right)\right)}^{⊤}{\left(T_{n}\left(b\right){\left(T_{n}\left(b\right)\right)}^{⊤}\right)}^{-1}T_{n}\left(a\right)\;\forall n\in N.$$

Applying Theorem 4.3 from [10] yields

$$\begin{array}{cc} \lambda_{n}\left(E\left(x_{n:1}x_{n:1}^{⊤}\right)\right) & =\frac{1}{{∥{\left(E\left(x_{n:1}x_{n:1}^{⊤}\right)\right)}^{-1}∥}_{2}}=\frac{1}{{∥\frac{1}{\sigma_{w}^{2}}{\left(T_{n}\left(a\right)\right)}^{⊤}{\left(T_{n}\left(b\right){\left(T_{n}\left(b\right)\right)}^{⊤}\right)}^{-1}T_{n}\left(a\right)∥}_{2}}\\ \; & \geq \frac{\sigma_{w}^{2}}{{∥(T_{n}\left(a\right))∥}_{2}^{2}{∥{\left(T_{n}\left(b\right)\right)}^{-1}∥}_{2}^{2}}\geq \frac{\sigma_{w}^{2}}{\max_{\omega \in [0,2\pi ]}{\left|a\left(\omega \right)\right|}^{2}K_{2}^{2}}>0\;\forall n\in N. \end{array}$$

Finally, we prove (11). From (A1) we need to show that $\left\{{∥E\left(x_{n:1}x_{n:1}^{⊤}\right)-C_{x_{n:1}}∥}_{F}\right\}$ is bounded. To that end, from Equation (16) in [11] we need to show that $\left\{∥E\left(x_{n:1}x_{n:1}^{⊤}\right)-T_{n}\left(\sigma_{w}^{2}\frac{{\left|b\right|}^{2}}{{\left|a\right|}^{2}}\right){∥}_{F}\right\}$ and $\left\{{∥T_{n}\left(\sigma_{w}^{2}\frac{{\left|b\right|}^{2}}{{\left|a\right|}^{2}}\right)-C_{n}\left(\sigma_{w}^{2}\frac{{\left|b\right|}^{2}}{{\left|a\right|}^{2}}\right)∥}_{F}\right\}$ are bounded. This is shown in the following two steps. *Step 1.* Using Lemma 4.2 and Theorem 4.3 from [10] we have:

Exn:1xn:1⊤−Tnσw2|b|2|a|2F=σw2Tn(a)−1Tn(b)Tn(b)⊤Tn(a)⊤−1−Tnσw2|b|2|a|2F≤Tn(a)−122σw2Tn(b)Tn(b)⊤−Tn(a)Tnσw2|b|2|a|2Tn(a)⊤F≤σw2K12Tn(b)Tn(b)⊤−Tn(a)Tn|b|2|a|2+Tn|b|2Tn1|a|2               −Tn|b|2Tn1|a|2Tn(a)⊤F≤σw2K12Tn(a)2Λ1Tn(a)⊤2Tn(b)Tn(b)⊤−Tn(a)Tn|b|2Tn1|a|2Tn(a)⊤F+Tn(b)Tn(b)⊤−Tn(a)Tn(|b|2)Tn1|a|2Tn(a)⊤F≤σw2K12Tn(a)22Λ1+Λ2Tn1|a|2F+Tn|b|2−Tn(a)Tn(|b|2)Tn1|a|2Tn(a)⊤F≤σw2K12Tn(a)22Λ1+Λ2+Tn|b|2−Tn(a)Tn|b|2Tn1aF+Tn(a)Tn|b|2Tn1a−Tn(a)Tn|b|2Tn1|a|2Tn(a)⊤F≤σw2K12Tn(a)22Λ1+Λ2+Λ3+Tn(a)2Tn|b|22Λ4+Λ5Tn1a2≤σw2K12maxω∈[0,2π]|a(ω)|2Λ1+Λ2+Λ3+maxω∈[0,2π]|a(ω)|maxω∈[0,2π]|b(ω)|2Λ4+Λ5minω∈[0,2π]|a(ω)|,

where

$$\begin{array}{cc} \Lambda_{1} & ={∥T_{n}\left({\left|b\right|}^{2}\right)T_{n}\left(\frac{1}{{\left|a\right|}^{2}}\right)-T_{n}\left(\frac{{\left|b\right|}^{2}}{{\left|a\right|}^{2}}\right)∥}_{F},\\ \Lambda_{2} & ={∥T_{n}\left(b\right){\left(T_{n}\left(b\right)\right)}^{⊤}-T_{n}\left({\left|b\right|}^{2}\right)∥}_{F},\\ \Lambda_{3} & ={∥T_{n}\left({\left|b\right|}^{2}\right)-T_{n}\left({a|b|}^{2}\right)T_{n}\left(\frac{1}{a}\right)∥}_{F},\\ \Lambda_{4} & ={∥T_{n}\left(\frac{1}{a}\right)-T_{n}\left(\frac{1}{{\left|a\right|}^{2}}\right)T_{n}\left(\bar{a}\right)∥}_{F},\\ \Lambda_{5} & ={∥T_{n}\left({a|b|}^{2}\right)-T_{n}\left(a\right)T_{n}\left({\left|b\right|}^{2}\right)∥}_{F}. \end{array}$$

From Lemma 2 in [13], $\left\{∥E\left(x_{n:1}x_{n:1}^{⊤}\right)-T_{n}\left(\sigma_{w}^{2}\frac{{\left|b\right|}^{2}}{{\left|a\right|}^{2}}\right){∥}_{F}\right\}$ is bounded. *Step 2.* Using Theorem 4.4, Lemmas 5.2 and 5.3 from [10] we have:

$$\begin{array}{cc} \; & {∥T_{n}\left(\sigma_{w}^{2}\frac{{\left|b\right|}^{2}}{{\left|a\right|}^{2}}\right)-C_{n}\left(\sigma_{w}^{2}\frac{{\left|b\right|}^{2}}{{\left|a\right|}^{2}}\right)∥}_{F}\\ \; & \leq \sigma_{w}^{2}\left(\Gamma_{1}+{∥T_{n}\left({\left|b\right|}^{2}\right)T_{n}\left(\frac{1}{{\left|a\right|}^{2}}\right)-C_{n}\left({\left|b\right|}^{2}\right)C_{n}\left(\frac{1}{{\left|a\right|}^{2}}\right)∥}_{F}\right)\\ \; & \begin{array}{c} \leq \sigma_{w}^{2}\left(\Gamma_{1}+{∥T_{n}\left({\left|b\right|}^{2}\right)T_{n}\left(\frac{1}{{\left|a\right|}^{2}}\right)-C_{n}\left({\left|b\right|}^{2}\right)T_{n}\left(\frac{1}{{\left|a\right|}^{2}}\right)∥}_{F}\right.\\ \left.+{∥C_{n}\left({\left|b\right|}^{2}\right)T_{n}\left(\frac{1}{{\left|a\right|}^{2}}\right)-C_{n}\left({\left|b\right|}^{2}\right)C_{n}\left(\frac{1}{{\left|a\right|}^{2}}\right)∥}_{F}\right) \end{array}\\ \; & \begin{array}{c} \leq \sigma_{w}^{2}\left(\Gamma_{1}+{∥T_{n}\left({\left|b\right|}^{2}\right)-C_{n}\left({\left|b\right|}^{2}\right)∥}_{F}{∥T_{n}\left(\frac{1}{{\left|a\right|}^{2}}\right)∥}_{2}\right.\\ \left.+{∥C_{n}\left({\left|b\right|}^{2}\right)∥}_{2}{∥T_{n}\left(\frac{1}{{\left|a\right|}^{2}}\right)-C_{n}\left(\frac{1}{{\left|a\right|}^{2}}\right)∥}_{F}\right) \end{array}\\ \; & \begin{array}{c} \leq \sigma_{w}^{2}\left(\Gamma_{1}+\Gamma_{2}{∥T_{n}\left(\frac{1}{{\left|a\right|}^{2}}\right)∥}_{2}+{∥C_{n}\left({\left|b\right|}^{2}\right)∥}_{2}\Gamma_{3}\right.\\ \left.+{∥C_{n}\left({\left|b\right|}^{2}\right)∥}_{2}{∥{\left(T_{n}\left({\left|a\right|}^{2}\right)\right)}^{-1}-C_{n}\left(\frac{1}{{\left|a\right|}^{2}}\right)∥}_{F}\right) \end{array}\\ \; & \begin{array}{c} \leq \sigma_{w}^{2}\left(\Gamma_{1}+\Gamma_{2}{∥T_{n}\left(\frac{1}{{\left|a\right|}^{2}}\right)∥}_{2}+{∥C_{n}\left({\left|b\right|}^{2}\right)∥}_{2}\Gamma_{3}\right.\\ \left.+{∥C_{n}\left({\left|b\right|}^{2}\right)∥}_{2}{∥{\left(T_{n}\left({\left|a\right|}^{2}\right)\right)}^{-1}∥}_{2}{∥I_{n}-T_{n}\left({\left|a\right|}^{2}\right){\left(C_{n}\left({\left|a\right|}^{2}\right)\right)}^{-1}∥}_{F}\right) \end{array}\\ \; & \begin{array}{c} \leq \sigma_{w}^{2}\left(\Gamma_{1}+\Gamma_{2}{∥T_{n}\left(\frac{1}{{\left|a\right|}^{2}}\right)∥}_{2}+{∥C_{n}\left({\left|b\right|}^{2}\right)∥}_{2}\Gamma_{3}\right.\\ \left.+{∥C_{n}\left({\left|b\right|}^{2}\right)∥}_{2}{∥{\left(T_{n}\left({\left|a\right|}^{2}\right)\right)}^{-1}∥}_{2}{∥C_{n}\left({\left|a\right|}^{2}\right)-T_{n}\left({\left|a\right|}^{2}\right)∥}_{F}{∥{\left(C_{n}\left({\left|a\right|}^{2}\right)\right)}^{-1}∥}_{2}\right) \end{array}\\ \; & \begin{array}{c} =\sigma_{w}^{2}\left(\Gamma_{1}+\Gamma_{2}{∥T_{n}\left(\frac{1}{{\left|a\right|}^{2}}\right)∥}_{2}+{∥C_{n}\left({\left|b\right|}^{2}\right)∥}_{2}\Gamma_{3}\right.\\ \left.+{∥C_{n}\left({\left|b\right|}^{2}\right)∥}_{2}{∥{\left(T_{n}\left({\left|a\right|}^{2}\right)\right)}^{-1}∥}_{2}\Gamma_{4}{∥{\left(C_{n}\left({\left|a\right|}^{2}\right)\right)}^{-1}∥}_{2}\right) \end{array}\\ \; & \leq \sigma_{w}^{2}\left(\Gamma_{1}+\frac{\Gamma_{2}}{\min_{\omega \in [0,2\pi ]}{\left|a\left(\omega \right)\right|}^{2}}+\max_{\omega \in [0,2\pi ]}{\left|b\left(\omega \right)\right|}^{2}\Gamma_{3}+\frac{\max_{\omega \in [0,2\pi ]}{\left|b\left(\omega \right)\right|}^{2}}{{\left(\min_{\omega \in [0,2\pi ]}{\left|a\left(\omega \right)\right|}^{2}\right)}^{2}}\Gamma_{4}\right), \end{array}$$

where

$$\begin{array}{cc} \Gamma_{1} & ={∥T_{n}\left(\frac{{\left|b\right|}^{2}}{{\left|a\right|}^{2}}\right)-T_{n}\left({\left|b\right|}^{2}\right)T_{n}\left(\frac{1}{{\left|a\right|}^{2}}\right)∥}_{F},\\ \Gamma_{2} & ={∥T_{n}\left({\left|b\right|}^{2}\right)-C_{n}\left({\left|b\right|}^{2}\right)∥}_{F},\\ \Gamma_{3} & ={∥T_{n}\left(\frac{1}{{\left|a\right|}^{2}}\right)-{\left(T_{n}\left({\left|a\right|}^{2}\right)\right)}^{-1}∥}_{F},\\ \Gamma_{4} & ={∥C_{n}\left({\left|a\right|}^{2}\right)-T_{n}\left({\left|a\right|}^{2}\right)∥}_{F}. \end{array}$$

From Lemmas 2 and 3 in [13] and Lemma 5.4 in [10], $\left\{{∥T_{n}\left(\sigma_{w}^{2}\frac{{\left|b\right|}^{2}}{{\left|a\right|}^{2}}\right)-C_{n}\left(\sigma_{w}^{2}\frac{{\left|b\right|}^{2}}{{\left|a\right|}^{2}}\right)∥}_{F}\right\}$ is bounded. □

## Appendix F. Proof of Theorem 6

**Proof.** Fix n>2m. From ([5] p. 1756) we have

$$\begin{array}{c} \frac{\sigma^{2}}{D}\left({\left(\frac{1}{n}\sum_{j=1}^{n}\sqrt{E\left({\left|y_{j}\right|}^{2}\right)}\right)}^{2}-{\left(\frac{1}{n}\sum_{j=1}^{n}\sqrt{\lambda_{j}\left(T_{n}\left(f\right)\right)}\right)}^{2}\right)\\ \leq \frac{2\sigma^{2}}{D\sqrt{n}}\sqrt{\frac{\max(f)}{\min(f)}}\sqrt{\sum_{k=1}^{m}k\left({\left|f_{k}\right|}^{2}+{\left|f_{-k}\right|}^{2}\right)}. \end{array}$$

Consequently, applying (1) and (8), we conclude that

$${\tilde{P}}_{n}\left(D\right)-P_{n}\left(D\right)\leq \frac{2\sigma^{2}}{D\sqrt{n}}\sqrt{\frac{\max(f)}{\min(f)}}\sqrt{\sum_{k=1}^{m}k\left({\left|f_{k}\right|}^{2}+{\left|f_{-k}\right|}^{2}\right)}.$$

□

## References

[1] Fresnedo O., Vazquez-Araujo F.J., Castedo L., Garcia-Frias J. (2013). Low-Complexity Near-Optimal Decoding for Analog Joint Source Channel Coding Using Space-Filling Curves. IEEE Commun. Lett..

[2] Sadhu V., Zhao X., Pompili D. (2020). Energy-Efficient Analog Sensing for Large-Scale and High-Density Persistent Wireless Monitoring. IEEE Internet Things J..

[3] Mouris B.A., Stavrou P.A., Thobaben R. (2022). Optimizing Low-Complexity Analog Mappings for Low-Power Sensors with Energy Scheduling Capabilities. IEEE Internet Things J..

[4] Lee K.H., Petersen D.P. (1976). Optimal Linear Coding for Vector Channels. IEEE Trans. Commun..

[5] Insausti X., Crespo P.M., Gutiérrez-Gutiérrez J., Zárraga-Rodríguez M. (2018). Low-Complexity Analog Linear Coding Scheme. IEEE Commun. Lett..

[6] Gutiérrez-Gutiérrez J., Villar-Rosety F.M., Zárraga-Rodríguez M., Insausti X. (2019). A Low-Complexity Analog Linear Coding Scheme for Transmitting Asymptotically WSS AR Sources. IEEE Commun. Lett..

[7] Gray R.M. (1972). On the Asymptotic Eigenvalue Distribution of Toeplitz Matrices. IEEE Trans. Inf. Theory.

[8] Gray R.M. (2006). Toeplitz and Circulant Matrices: A review. Found. Trends Commun. Inf. Theory.

[9] Gutiérrez-Gutiérrez J., Zárraga-Rodríguez M., Villar-Rosety F.M., Insausti X. (2018). Rate-Distortion Function Upper Bounds for Gaussian Vectors and Their Applications in Coding AR Sources. Entropy.

[10] Gutiérrez-Gutiérrez J., Crespo P.M. (2011). Block Toeplitz Matrices: Asymptotic Results and Applications. Found. Trends Commun. Inf. Theory.

[11] Zárraga-Rodríguez M., Gutiérrez-Gutiérrez J., Insausti X. (2019). A Low-Complexity and Asymptotically Optimal Coding Strategy for Gaussian Vector Sources. Entropy.

[12] Gutiérrez-Gutiérrez J., Crespo P.M. (2011). Asymptotically Equivalent Sequences of Matrices and Multivariate ARMA Processes. IEEE Trans. Inf. Theory.

[13] Gutiérrez-Gutiérrez J., Zárraga-Rodríguez M., Insausti X. (2020). On the Asymptotic Optimality of a Low-Complexity Coding Strategy for WSS, MA, and AR Vector Sources. Entropy.

